# Embolic Stroke Due to Symptomatic Nonstenotic Carotid Disease

**DOI:** 10.7759/cureus.104982

**Published:** 2026-03-10

**Authors:** Bilal A Darwish, Hussam Yacoub

**Affiliations:** 1 Department of Neurology, Lehigh Valley Health Network, Allentown, USA

**Keywords:** carotid artery, ischemic stroke, low-grade stenosis, ulcerated plaque, vulnerable plaque

## Abstract

Atherosclerosis of the internal carotid artery (ICA) can lead to clinically significant stenosis. Cerebral vascular events are often caused by plaque rupture and embolization. However, there are options in the management of symptomatic, low-grade CAS (<50%), with vulnerable plaque features.

An 80-year-old man presented with an acute onset of right-sided weakness. Neurologic examination revealed pronounced right lower-extremity weakness. Head computed tomography (CT) showed no acute hemorrhage or ischemia. CT angiogram (CTA) of the head and neck revealed an ulcerated plaque with 40% stenosis of the left carotid bifurcation. Magnetic resonance imaging (MRI) of the brain showed scattered infarcts in the territory of the left anterior cerebral artery. The patient was deemed an ideal candidate for carotid revascularization and underwent endarterectomy with no complications. He was discharged home with no recurrence of stroke.

A 79-year-old man presented to the emergency department with an acute onset of right arm weakness and incoordination. Head CT showed chronic-appearing infarcts in the left paracentral gyri, corona radiata, and centrum semiovale. A CTA of the head and neck revealed an ulcerated plaque of the left cervical ICA origin with 45-50% stenosis per NASCET criteria. MRI of the brain showed multifocal acute left cerebral infarcts in a linear distribution, suggesting watershed territory ischemia. The patient was deemed an ideal candidate for revascularization of the left common carotid/ ICA for stroke prevention and underwent endarterectomy. He was discharged home with no recurrence of stroke.

Our two cases emphasize the importance of considering ICA with vulnerable plaque as a source of embolic stroke, regardless of the degree of stenosis, and the benefit of revascularization for stroke prevention.

## Introduction

The etiology of ischemic stroke is classified into large artery atherosclerosis, cardioembolic, small vessel disease, acute stroke of other determined etiology, and acute stroke of undetermined source. According to the Trial of Org 10172 in acute stroke treatment (TOAST) criteria, large artery atherosclerosis is defined by the degree of stenosis [1]. The definition includes patients with carotid or other extracranial/intracranial lesions with ≥50% stenosis, as defined in the North American Symptomatic Carotid Endarterectomy Trial (NASCET) [2].

Internal carotid artery (ICA) stenosis contributes to 7% of all ischemic strokes. Advanced age, hypertension, tobacco use, and diabetes are common risk factors that can contribute to plaque formation and, consequently, atherosclerosis. Rupture and embolization of vulnerable, unstable plaque can lead to acute ischemic stroke, despite the degree of stenosis. Clinical features vary and include transient visual or neurological symptoms [3].

Carotid artery stenosis (CAS) is diagnosed by carotid duplex ultrasound, computed tomography angiography (CTA), or magnetic resonance angiography. The current gold standard is digital subtraction angiography [4].

Embolic stroke of undetermined source (ESUS) accounts for 9%-25% of ischemic strokes, and 4.5% of strokes recur despite optimal medical management. The introduction of improved imaging and prolonged cardiac monitoring improves the classification of cardioembolic stroke or paradoxical emboli due to patent foramen ovale. Thrombectomy specimens have revealed high platelet content in numerous patients with cryptogenic, embolic-appearing stroke, suggesting vessel-to-vessel embolization as an etiology [5].

Previous studies have described an association between specific plaque features and the risk of embolization, regardless of the degree of stenosis, particularly in patients with ESUS [6]. Intraplaque hemorrhage (IPH) is a key feature of plaque vulnerability that contributes to lipid-rich necrotic core enlargement and plaque progression [7]. Other features of carotid plaque vulnerability include a lipid-rich necrotic core, alterations in the fibrous cap, and inflammation [7-9]. Nonstenotic carotid plaques are commonly seen on the side ipsilateral to stroke in patients with ESUS. Therefore, symptomatic nonstenotic carotid (SyNC) disease may play a role in the etiology of embolic stroke.

The precise definition of SyNC is unclear. The most widely accepted criteria for making the diagnosis of SyNC include: 1) carotid plaque associated with < 50% stenosis, 2) plaque with high-risk features that change morphology over time, 3) recurrent embolic stroke in the ipsilateral ICA territory, and 4) absence of any other cause of stroke [6]. The NASCET and European Carotid Surgery Trial recommend revascularization in patients with symptomatic, moderate (50-69%) or severe (70-99%) CAS, with a life expectancy of >2 years and a <6% risk of morbidity and mortality [2,10]. Revascularization of the ICA with low-grade stenosis is not typically recommended [11], and optimal management of asymptomatic moderate CAS for stroke prevention remains unidentified. However, improved strategies for stroke prevention in patients with ICA-vulnerable plaque are warranted, given the high risk of embolization independent of the degree of stenosis.

## Case presentation

Case 1

An 80-year-old man presented to the emergency department with an acute onset of right-sided weakness. The patient had a past medical history of hypertension, hyperlipidemia, and coronary artery disease. Family and social history were not pertinent. Prescribed medications included oral aspirin 81 mg daily, a statin, and anti-hypertensive agents. The patient was afebrile, with a blood pressure of 132/80 mmHg, heart rate of 88 bpm, respiratory rate of 24 breaths per minute, and oxygen saturation of 95%. Initial neurological examination revealed heel-to-shin ataxia on the right. Language, motor, and sensory examination was unrevealing.

Complete blood count and comprehensive metabolic panel were within normal limits. Low-density lipoprotein was 65 mg/dL, and hemoglobin A1c was 5.9% (Table 1).

**Table 1:** Case 1: Complete metabolic panel, lipid panel, hemoglobin A1c%, and complete blood count HDL: high-density lipoprotein; LDL: low-density lipoprotein; BUN: blood urea nitrogen; eGFR: estimated glomerular filtration rate; MPV: mean platelet volume; MCV: mean corpuscular volume; MCH: mean corpuscular hemoglobin; MCHC: mean corpuscular hemoglobin concentration; RDW: red blood cell distribution width; AST: aspartate aminotransferase; ALT: alanine aminotransferase

Laboratory Parameter	Value	Units	Reference Range
Glucose	111	mg/dL	65–99
BUN	17	mg/dL	7–28
Creatinine	0.83	mg/dL	0.53–1.30
eGFR	88	mL/min/1.73m²	>59
Sodium	135	mmol/L	135–145
Potassium	3.7	mmol/L	3.5–5.2
Chloride	101	mmol/L	100–109
CO₂	28	mmol/L	21–31
Calcium	9.8	mg/dL	8.5–10.1
Anion Gap	6		3–11
Alkaline Phosphatase	37	U/L	35–120
AST	19	U/L	<41
ALT	28	U/L	<56
Total Bilirubin	0.7	mg/dL	0.2–1.0
Albumin	4.2	g/dL	3.5–5.7
Total Protein	6.8	g/dL	6.3–8.3
Total Cholesterol	127	mg/dL	<200
Triglycerides	136	mg/dL	<150
HDL Cholesterol	35	mg/dL	23–92
LDL Cholesterol (calculated)	65	mg/dL	<130
Non-HDL Cholesterol	92	mg/dL	<160
Cholesterol/HDL Ratio	3.6		
Hemoglobin A1c	5.9	%	<5.7
Estimated Average Glucose	123	mg/dL	
Hemoglobin	13.9	g/dL	12.5–17.0
Hematocrit	40.3	%	37.0–48.0
WBC	8.1	×10³/µL	4.0–10.5
RBC	4.45	×10⁶/µL	4.00–5.40
Platelets	206	×10³/µL	140–350
MCV	91	fL	80–100
MCH	31.2	pg	27.0–36.0
MCHC	34.4	g/dL	32.0–37.0
RDW	12.7	%	12.0–16.0
MPV	8.2	fL	7.5–11.3
Absolute Neutrophils	6.1	×10³/µL	1.8–7.8
Absolute Lymphocytes	1.1	×10³/µL	1.0–3.0
Absolute Monocytes	0.8	×10³/µL	0.3–1.0
Absolute Eosinophils	0.1	×10³/µL	0.0–0.5
Absolute Basophils	0	×10³/µL	0.0–0.1
Neutrophils	75	%	
Lymphocytes	14	%	
Monocytes	9	%	
Eosinophils	1	%	
Basophils	1	%	

**Figure 1:**
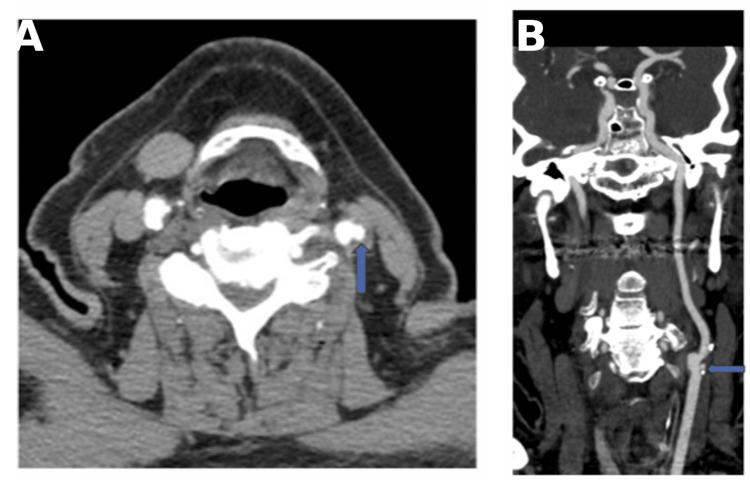
Case 1: A (axial) and B (coronal) reconstruction CTA revealing a soft plaque at the left carotid bifurcation with a 5 mm plaque ulceration (arrows) and approximately 40% stenosis by the NASCET criteria. CTA: CT angiogram; NASCET: North American Symptomatic Carotid Endarterectomy Trial

**Figure 2:**
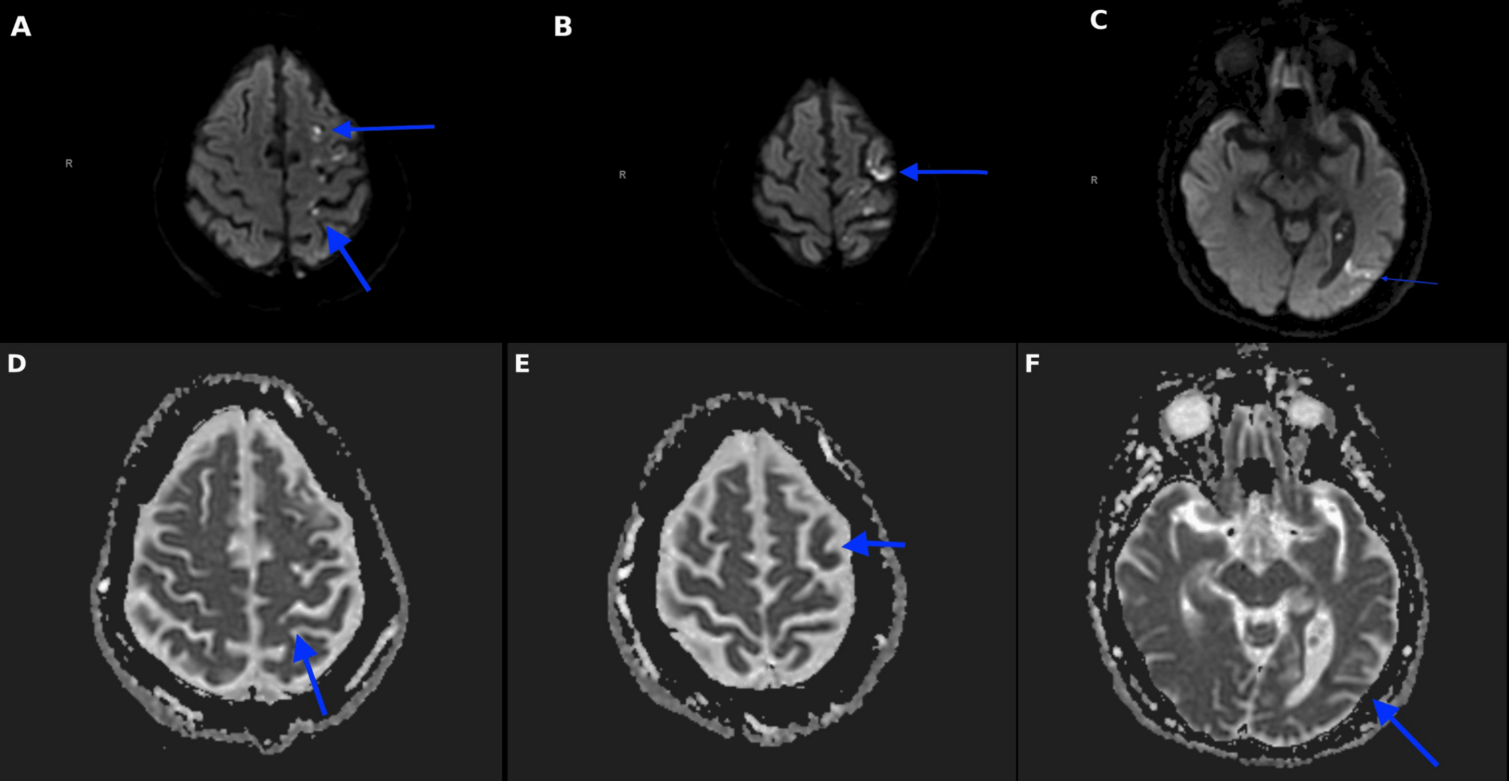
Case 1: (A-F) Numerous areas of restricted diffusion involving the left frontal lobe, parietal lobe, and left MCA/PCA watershed territory with an ADC correlate consistent with an infarct (A-C) Axial MRI brain diffusion weighted imaging (DWI); (D-F) Axial MRI brain apparent diffusion coefficient (ADC) MCA: middle cerebral artery; PCA: posterior cerebral artery

**Figure 3:**
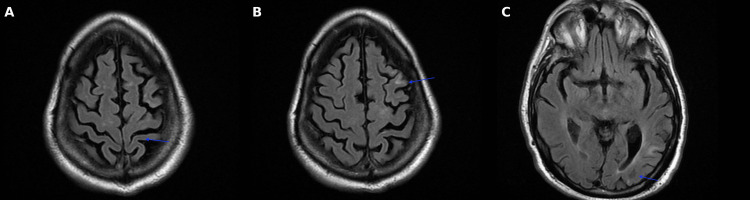
Case 1: (A-C) Corresponding axial MRI brain FLAIR sequence showing the corresponding areas of restricted diffusion seen in the previous figure. FLAIR: fluid attenuated inversion recovery

**Figure 4:**
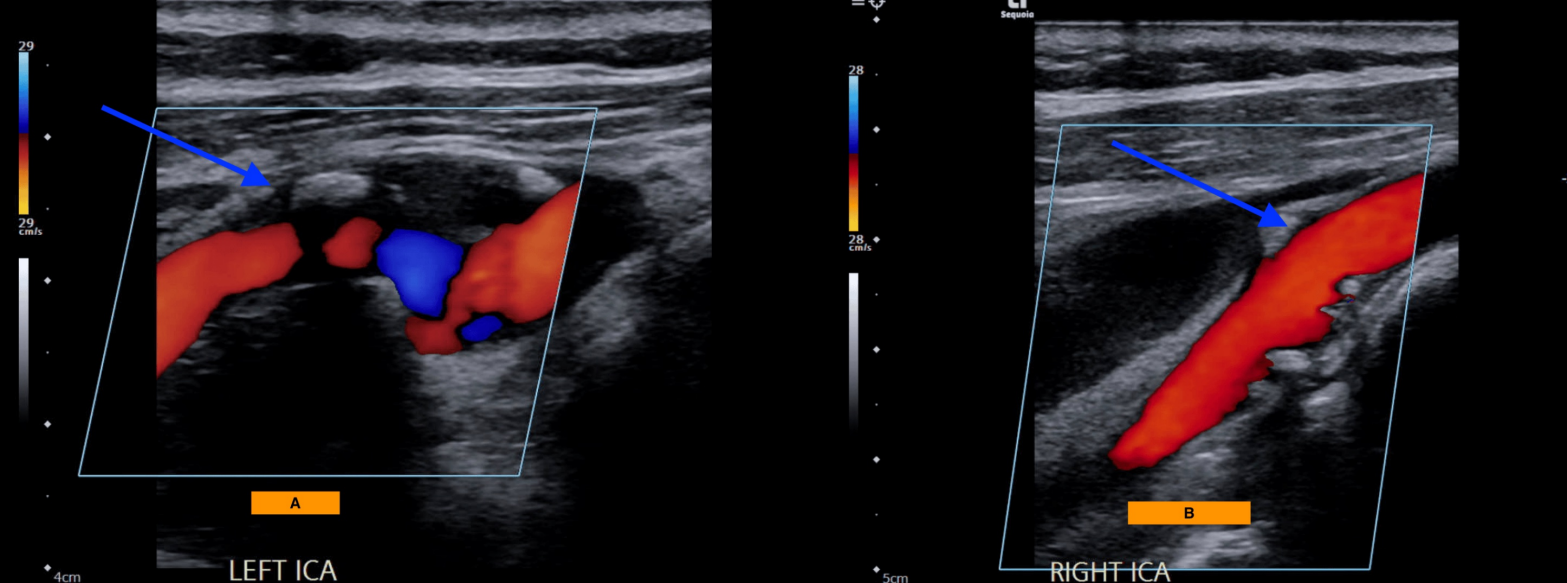
Case 1: Carotid artery ultrasound (A) <50% left ICA stenosis, heterogeneous plaque present (B) <50% right ICA stenosis, heterogeneous plaque present ICA: internal carotid artery

CT of the head showed no evidence of intracerebral hemorrhage. CTA of the head and neck revealed a soft plaque in the left carotid bifurcation with a 5 mm plaque ulceration (Figures 1A, 1B) and approximately 40% stenosis based on the NASCET criteria. Magnetic resonance imaging (MRI) of the brain showed scattered infarcts in the left anterior cerebral artery territory (Figures 2A-2F, 3A-3C). Carotid artery ultrasound revealed <50% stenosis of the bilateral ICAs based on velocity criteria, with sonographic evidence of ulcerated plaque at the left ICA bifurcation (Figures 4A, 4B).

Further workup for embolic stroke included a 2D-echocardiogram, which showed a grade I diastolic dysfunction, but no evidence of intracardiac thrombus. Subsequently, the patient underwent a transesophageal echocardiogram, which showed a normal left atrial appendage and no thrombus in the left atrial cavity. Telemetry monitoring revealed no evidence of arrhythmia. The patient was started on intravenous heparin and aspirin 81 mg daily. Vascular surgery was consulted, and after coordinating with vascular neurology, the patient was deemed an ideal candidate for revascularization of the left common carotid/ICA for stroke prevention. He underwent an endarterectomy with bovine patch angioplasty with no complications and no recurrence of emboli. He was ultimately discharged home on aspirin 81 mg daily and a statin.

Case 2

A 79-year-old man presented to the emergency department with an acute onset of right-arm weakness and incoordination. The patient had a past medical history of hypertension, diabetes mellitus type 2, and a left-sided subcortical infarct. Family and social history were not pertinent. Prescribed medications included oral aspirin 81 mg daily, clopidogrel 75 mg daily, a statin, metformin, empagliflozin, and anti-hypertensive agents.

The patient was afebrile with a blood pressure of 142/92 mmHg, heart rate of 75 bpm, respiratory rate of 17 breaths per minute, and oxygen saturation of 97%. Initial neurological examination revealed right-sided ataxia on finger-to- nose testing, mild right-sided sensory loss to light touch, and extinction to the right side. Language and motor examination were unrevealing.

Complete blood count and comprehensive metabolic panel were within normal limits. Low-density lipoprotein was 109 mg/dL, and hemoglobin A1c was 8.6% (Table 2).

**Table 2:** Case 2: Complete metabolic panel, lipid panel, hemoglobin A1c%, and complete blood count HDL: high-density lipoprotein; LDL: low-density lipoprotein; BUN: blood urea nitrogen; eGFR: estimated glomerular filtration rate; MPV: mean platelet volume; MCV: mean corpuscular volume; MCH: mean corpuscular hemoglobin; MCHC: mean corpuscular hemoglobin concentration; RDW: red blood cell distribution width; AST: aspartate aminotransferase; ALT: alanine aminotransferase

Laboratory Parameter	Value	Units	Reference Range
Hemoglobin A1c	8.6	%	<5.7
Estimated Average Glucose (eAG)	200	mg/dL	
Total Cholesterol	184	mg/dL	<200
Triglycerides	123	mg/dL	<150
HDL Cholesterol (Direct)	50	mg/dL	23–92
Non-HDL Cholesterol	134	mg/dL	<160
LDL Cholesterol (Calculated)	109	mg/dL	<130
Cholesterol/HDL Ratio	3.7		
Hemoglobin	10.9	g/dL	12.5–17.0
Hematocrit	31.6	%	37.0–48.0
WBC	6.1	×10³/µL	4.0–10.5
RBC	3.67	×10⁶/µL	4.00–5.40
Platelet Count	153	×10³/µL	140–350
MPV	9.5	fL	7.5–11.3
MCV	86	fL	80–100
MCH	29.8	pg	27.0–36.0
MCHC	34.5	g/dL	32.0–37.0
RDW	13.4	%	12.0–16.0
Absolute Neutrophils	4	×10³/µL	1.8–7.8
Absolute Lymphocytes	1.5	×10³/µL	1.0–3.0
Absolute Monocytes	0.5	×10³/µL	0.3–1.0
Absolute Eosinophils	0.1	×10³/µL	0.0–0.5
Absolute Basophils	0	×10³/µL	0.0–0.1
Neutrophils	64	%	
Lymphocytes	24	%	
Monocytes	9	%	
Eosinophils	2	%	
Basophils	1	%	
Glucose	105	mg/dL	65–99
BUN	19	mg/dL	7–28
Creatinine	0.83	mg/dL	0.53–1.30
Sodium	139	mmol/L	135–145
Potassium	3.6	mmol/L	3.5–5.2
Chloride	106	mmol/L	100–109
Carbon Dioxide (CO₂)	24	mmol/L	21–31
Calcium	9.4	mg/dL	8.5–10.5
Alkaline Phosphatase	36	U/L	35–120
Albumin	4.2	g/dL	3.5–5.7
Total Bilirubin	0.6	mg/dL	0.2–1.0
Total Protein	6.7	g/dL	6.3–8.3
AST	18	U/L	<41
ALT	16	U/L	<56
Anion Gap	9		3–11
eGFR (creatinine)	89	mL/min/1.73m²	>59

**Figure 5:**
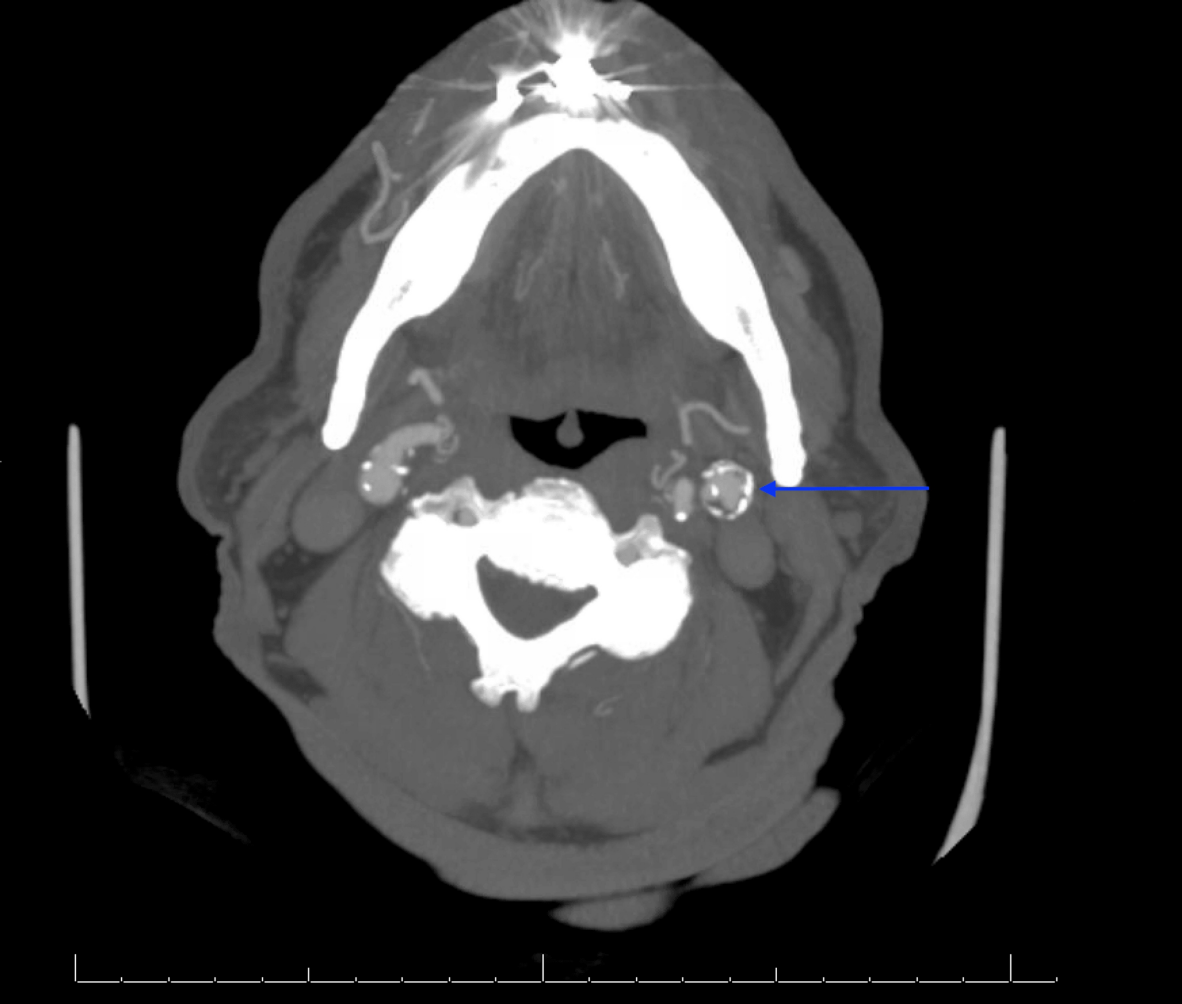
Axial CTA head/neck revealing an ulcerated plaque of the left cervical ICA origin (arrow) with 45-50% stenosis per NASCET criteria CTA: CT angiogram; ICA: internal carotid artery; NASCET: North American Symptomatic Carotid Endarterectomy Trial

**Figure 6:**
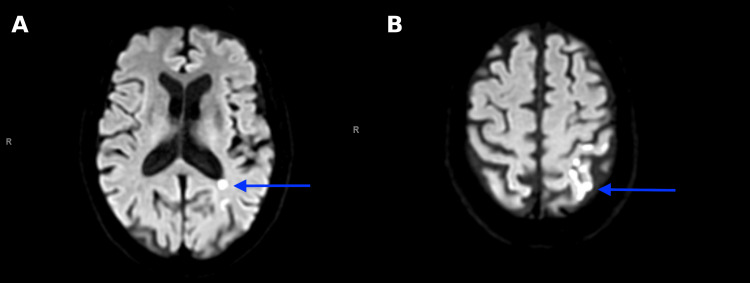
MRI brain axial diffusion weighted imaging sequence (A) Area of restricted diffusion at the periatrial region of the left lateral ventricle; (B) Areas of restricted diffusion at the left parietal lobe

CT of the head showed chronic-appearing infarcts in the left paracentral gyri, left corona radiata, and centrum semiovale, with no evidence of intracerebral hemorrhage. A CTA of the head and neck revealed an ulcerated plaque of the left cervical ICA origin with 45-50% stenosis per NASCET criteria (Figure 5). MRI of the brain showed multifocal acute left cerebral infarcts in a linear distribution (Figures 6A, 6B), suggesting watershed territory ischemia.

Further workup for embolic stroke included 2D echocardiography, which showed Grade I diastolic dysfunction and an ejection fraction of 55%, but no evidence of intracardiac thrombus. Telemetry monitoring revealed no evidence of arrhythmia.

The patient was loaded with clopidogrel 300 mg once and subsequently continued on clopidogrel 75 mg and aspirin 81 mg daily. Vascular surgery was consulted, and after coordinating with vascular neurology, the patient was deemed an ideal candidate for revascularization of the left common carotid/ICA for stroke prevention. He underwent a trans-carotid artery revascularization procedure with no complications and no recurrence of emboli. Following the procedure, the patient was discharged home on daily aspirin 81 mg, clopidogrel 75 mg daily, and a statin.

## Discussion

Severe ICA stenosis is a well-defined risk of embolic stroke, but the risk with nonstenotic, vulnerable plaques is increasingly identified. Advanced imaging techniques can be utilized to evaluate a subset of carotid artery plaques with features of vulnerability, such as ulceration or IPH.

A comprehensive systematic review and meta-analysis investigated the incidence or recurrence rate of transient ischemic attack (TIA) or acute ischemic stroke in patients with nonstenotic carotid plaque [12]. The risk of TIA/stroke recurrence was higher than the incidence of the first-ever event, and more than doubled when IPH was detected on imaging studies [13].

Another study evaluated the number of ischemic stroke cases that might be reclassified if taking into consideration ipsilateral, nonstenotic ICA plaque with high-risk features such as ulceration, IPH, or fibrous cap [13]. Approximately 20% of patients initially diagnosed with cryptogenic stroke were reclassified to having stroke related to large vessel atherosclerosis [13].

It is essential to consider revascularization in patients with vulnerable ICA plaque and ipsilateral embolic infarcts with no other source identified [7]. Studies showed that the risk of recurrent stroke in cases of <50% stenotic ICAs with vulnerable plaque is higher than the risk associated with asymptomatic ICAs with severe stenosis [8]. Kurosaki et al. evaluated 123 patients with symptomatic ICAs with low-grade stenosis and found the incidence of IPH to be 60% [9]. Other studies have shown carotid endarterectomy (CEA) to be safe and effective in patients with ulcerated plaque of the ICA and low-grade stenosis [7]. Ballota et al. found that survival rates after 1 year and 3 years were 98% and 90%, respectively, with no recurrent neurological events or late carotid occlusion [8]. A systematic review by Larson et al. analyzed six prospective and retrospective cohorts of patients with symptomatic, nonstenotic carotids. Of the 138 patients who underwent CEA, none had recurrent ipsilateral ischemic stroke. Twenty-eight patients from three studies included in this analysis were identified to have IPH on MRI prior to CEA, with no recurrence of stroke following revascularization. Mean follow-up after CEA was approximately 24 months [14].

Improved techniques in the setting of CAS may be essential for predicting stroke risk. Advanced imaging studies help characterize and detect plaque with high-risk features. A percentage of patients previously classified as ESUS had vulnerable plaque features not identified on conventional imaging studies [14]. Adjusting the current guidelines for focusing on vulnerable plaque features as an alternative to luminal stenosis alone could be beneficial in improving stroke prevention.

Further investigation in randomized clinical trials is warranted to verify the precise role of unstable carotid plaque in the management and prevention of ischemic stroke in patients with SyNC.

## Conclusions

We present two cases of patients with symptomatic ICA with vulnerable, ulcerated plaque and <50% stenosis. We highlight the importance of considering the carotid artery with vulnerable plaque as a source of embolic stroke, regardless of the degree of stenosis. Clinicians should be familiar with the radiographic features of unstable carotid artery plaque morphology and high risk of rupture. Revascularization of the carotid artery appears superior to medical management in this patient population, although further studies are warranted.
